# Glycoside Hydrolases across Environmental Microbial Communities

**DOI:** 10.1371/journal.pcbi.1005300

**Published:** 2016-12-19

**Authors:** Renaud Berlemont, Adam C. Martiny

**Affiliations:** 1 Dept. of Biological Sciences, California State University, Long Beach, California, United States of America; 2 Dept. of Earth System Science, University of California, Irvine, California, United States of America; 3 Dept. of Ecology and Evolutionary Biology, University of California, Irvine, California, United States of America; Institute of Microbiology of the ASCR, Prague, CZECH REPUBLIC

## Abstract

Across many environments microbial glycoside hydrolases support the enzymatic processing of carbohydrates, a critical function in many ecosystems. Little is known about how the microbial composition of a community and the potential for carbohydrate processing relate to each other. Here, using 1,934 metagenomic datasets, we linked changes in community composition to variation of potential for carbohydrate processing across environments. We were able to show that each ecosystem-type displays a specific potential for carbohydrate utilization. Most of this potential was associated with just 77 bacterial genera. The GH content in bacterial genera is best described by their taxonomic affiliation. Across metagenomes, fluctuations of the microbial community structure and GH potential for carbohydrate utilization were correlated. Our analysis reveals that both deterministic and stochastic processes contribute to the assembly of complex microbial communities.

## Author Summary

The deconstruction of complex carbohydrates (e.g., cellulose, chitin), mostly by microbes, releases short metabolizable oligosaccharides to the environment. This contributes to the functioning of an ecosystem and is essential for global carbon cycling. Carbohydrate degradation requires the production of carbohydrate active enzymes (CAZymes). Among these, GH are the most abundant enzymes to break down polysaccharides into smaller products. However, not all the microbes have genes for all the glycoside hydrolases (GH). In addition, microbial communities are dynamic assemblages and display important spatio-temporal variations. Thus, two major questions are, which microbes are associated with GH genes and which are involved in carbohydrate processing across environments. The bioinformatic challenge is therefore to collect enough metagenomic datasets and to reanalyze microbiomes in the light of GH genes. Here, we created a custom bioinformatic pipeline aimed at identifying sequences for GH in 1,934 sequenced microbiomes derived from 13 broadly defined ecosystems, including terrestrial and marine ecosystems as well as human and animal associated microbiomes. We linked changes in microbial community composition and functional potential for carbohydrate processing across environments. Our results suggest that a relatively small number of bacterial genera (i.e., the potential degraders), with increased number of GH genes target the substrates expected in their environment. These degraders display mostly conserved GH content across microbiomes. In each ecosystem however, the functional redundancy among potential degraders allows for slightly distinct communities with similar functional potential. Globally, linking variations of microbial community structure and function, across ecosystems, provides insight into how microbial communities may adjust to the supply of carbohydrates. In the future, this will help predict how change in microbial community composition, in response to environmental perturbation (e.g., global change), can affect the functional potential of microbial communities.

## Introduction

The complete enzymatic deconstruction of polysaccharides (e.g., cellulose, chitin) involves many carbohydrate active enzymes (CAZymes) including glycoside hydrolases (GH), polysaccharide lyases, carbohydrate esterases, accessory activities (e.g., LPMO), and many accessory domains (e.g., CBM)[1–4]. The glycoside hydrolases (GH) cleave glycosidic bonds in polysaccharides (e.g., cellulose) and oligosaccharides (e.g., cellooligosaccharides) and release short metabolizable products (e.g., cellobiose). According to the CAZy database [5], many GH families, identified based on their structure, display substrate specificity. For example, most biochemically characterized proteins with domains from GH families 5, 6, 7, 8, 9, 12, 44, 45, and 48 act on cellulose. On the other hand, some GH families display mixed substrate specificity (e.g., GH16). The identification of specific GH domains in sequenced genomes [6] and metagenomes [7] allows for the prediction of the potential for starch, cellulose, xylan, fructan, chitin, and dextran deconstruction (i.e., the potential to target carbohydrates according to functional annotation of genes)[2,6,8,9].

To date, most identified GH are from bacteria and their distribution, across sequenced genomes, is phylogeneticaly conserved within genera [2,9,10]. Most bacteria have the potential to target starch and oligosaccharides and few lineages are associated with increased potential for complex carbohydrate deconstruction (i.e., potential polysaccharide degraders) [2,9]. Besides some well-characterized microbial lineages involved in polysaccharide deconstruction (e.g., *Clostridium*, *Streptomyces*), the systematic investigation of sequenced bacterial genomes has revealed the richness and diversity of GH in poorly-characterized degrader lineages (e.g., *Actinospica*)[6].

Microbial communities exposed to varying parameters, including carbohydrate supply [11], fluctuate across environments [12–16]. As a consequence, changes in community composition have been associated with variations of environmental processes (e.g., plant material deconstruction, phosphate uptake) [17–19]. Thus, the major challenges are (i) to understand which bacteria are involved in carbohydrate deconstruction, and (ii) to understand if the overall microbial community composition and potential for carbohydrate deconstruction are linked, across microbial populations and across environments. Does the environment select for specific GH, specific lineages, or both [10,20]? In the first case, microbial communities would adapt through selection of adequate potential for carbohydrate processing independently of the lineage (e.g., by lateral gene transfer or other ways of convergent evolution). In the second hypothesis, microbial communities would adapt through selection of phylogenetically defined lineages endowed with specific potential for carbohydrate processing [20]. The first hypothesis implies that changes in functional potential and community composition are not connected whereas the opposite is the case for the alternative hypothesis. In order to address these questions, we investigated how changes in the potential for carbohydrate processing correlates with the change of bacterial communities composition across 13 broadly defined environments and across 1,934 sequenced microbiomes.

Despite the lack of consistent quantitative estimation of the carbohydrate composition across environments, ecosystem-types are associated with specific supplies of carbohydrates. In soil [21], sludge and wastewater (referred to as sludge below) [22], and in the phyllosphere [23], microbes are exposed to an abundant—and varying—complex mixture of carbohydrates (e.g., cellulose, xylan, and fructan from plant material and chitin from fungi and arthropods). In aquatic systems (i.e., marine, mats, and larger fresh water environments), the carbohydrate supply is reduced, and chitin is the most common polymer [24–26]. Microbes in digestive tracts (i.e., human gut, oral, and most animal samples) are exposed to diverse and abundant substrates including plant polysaccharides and animal glycosaminoglycans found in food and produced by the host [27–29]. In other parts of the host (e.g., skin), the supply of carbohydrates is reduced and mostly composed of animal carbohydrates [30]. In corals and sponges, the supply of carbohydrates is reduced and reflects the chemical composition of prey (i.e., detritus and planktonic cells)[31]. Finally, starch and glycogen, produced to store energy by many organisms [32,33], and dextran associated with bacterial biofilm (e.g., dental plaque) [34] are expected to be present in most environments.

Investigating how changes of microbial community composition and changes of potential for carbohydrate processing correlate across environments will (i) help identify environment-specific potential for carbohydrate processing, (ii) and highlight new environmental lineages associated with potential for carbohydrate utilization, and (iii) provide a comprehensive framework for the interpretation of the mechanisms by which microbial communities adapt to varying carbohydrate supply.

## Results and Discussion

### Glycoside hydrolases identification

**Fig 1:**
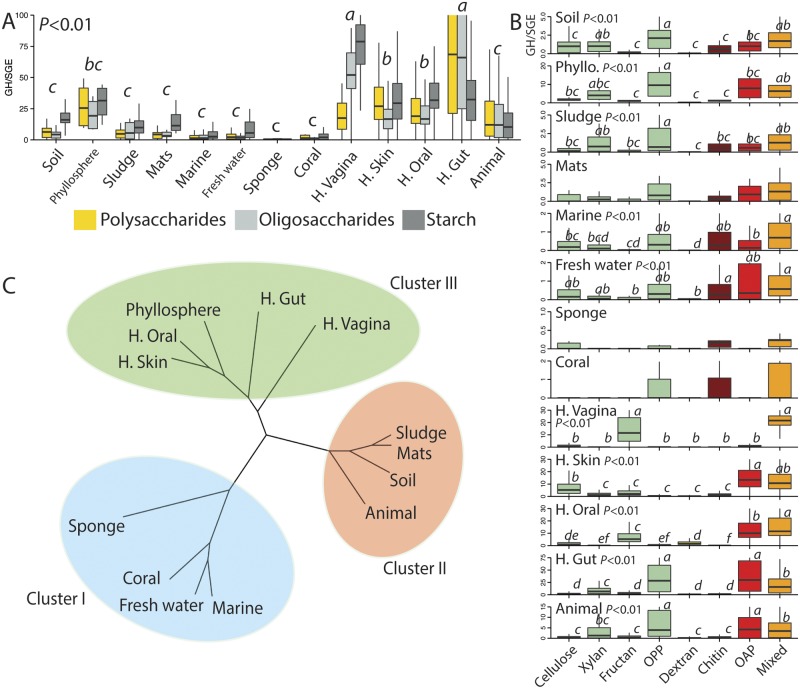
A and B, frequency, per sequenced genome equivalent (SGE), of sequences for GH across environments. Polysaccharides are cellulose, xylan, fructan, other plant polysaccharides (OPP), chitin, dextran, other animal polysaccharides (OAP), and mixed substrates; Starch stands for both starch and glycogen. *P*-values are from the overall ANOVA on square-root transformed data (*P*>0.05, Tukey post-hoc test). C, environments clustering according to the frequency (median) of identified sequences for each GH families, across ecosystem-types.

First, in order to test how the environment affected the potential for carbohydrate utilization across ecosystems, we identified 130.2×10^6^ sequences encoding putative glycoside hydrolases (GH, ~0.5% of analyzed sequences) in 1,934 annotated metagenomes from 13 broadly defined ecosystems (S1 Table) [35]. Across environments, we found that the potential for carbohydrate utilization varied extensively but, in many cases, matched the expected supply of carbohydrates. The frequency of sequences for GH ranged from 1.7 (sponges) to 172 (human gut) per sequenced genome equivalent (i.e., 3Mbp, SGE) [7,36]. Broadly, the overall frequency of identified GH was high in most human—associated ecosystems, intermediate in the phyllosphere and animal samples and low in soil, sludge, mats, marine, fresh-water, coral, and sponge samples (Fig 1A, S2 Table). Besides enzymes for oligosaccharides and starch, sequences targeting mixed substrates [i.e., the other plant polysaccharides (OPP), the other animal polysaccharides (OAP), and other undefined carbohydrate (Mixed)] dominated in most samples (Fig 1B, S3 Table). Next, sequences for cellulose and fructan utilization were abundant in most human samples, intermediate in the phyllosphere and soil and low in the other ecosystem types. Xylanases were abundant in the human gut and intermediate in animal and phyllosphere samples only. Chitinases were abundant in mats and human skin samples whereas sequences for dextran utilization were abundant in human mouth and gut. Environments with expected abundant and diverse supply of carbohydrates (e.g., human gut, animal, phyllosphere, soil) were associated with sequences for GH targeting many different substrates. Furthermore, the potential for carbohydrate processing was skewed in environments with a specific carbohydrate supply. In aquatic environments and the human mouth, the relative frequencies of sequences for GH targeting chitin and dextran were found to be higher than in other ecosystems, respectively (Fig 1B, S3 Table). In some environments however (e.g., human skin and vagina), the prevalence of sequences for GH targeting specific substrates (e.g., cellulose and fructan) did not systematically matched with the expected presence of substrates.

When accounting for both the presence/absence and frequency of sequences for GH, across ecosystem-types we observed three clusters (Fig 1C). The first cluster contained metagenomes from aquatic environments, sponge, and coral samples. In these ecosystems, the frequency of GH was extremely reduced. The second cluster contained metagenomes from soil, sludge, mats, and—more distantly related- animal samples. These ecosystems displayed intermediate and diverse GH frequency. Finally, the third group, composed of human samples and the phyllosphere, displayed abundant and diverse GH.

Globally each ecosystem-type displays a specific potential for polysaccharide deconstruction matching the assumed carbohydrate supply. Sequences for GH were more frequent in human, animal, and phyllosphere samples than in “open” environments. These fluctuations could reflect variations in the actual GH abundance and/or variations of the average genomes size across environments. Indeed, for example, many lineages derived from the soil have large genomes (e.g., *Streptomyces*, phylum Actinobacteria) whereas many host associated microbes have smaller genomes (e.g., *Mycobacterium*, phylum Actinobacteria) [37,38].

Within ecosystems, extensive variations were also observed. These variations, likely reflect environmental fluctuation in microbial community composition [e.g., human microbiome [39], animals [27], soil [40], and marine ecosystems [41]] in response to specific environmental conditions (e.g., moisture, carbohydrate supply) in sub-ecosystem types. For example “soil” represents many types of ecosystems (e.g., desert and forest) associated with distinct carbohydrate supply and host to different communities [11]. Alternatively, these variations could reflect the variable GH content among functionally equivalent, and potentially interchangeable, lineages. For example, not all the potential cellulose degraders display the same GH content [6].

### Identification of potential carbohydrate degrader lineages

**Fig 2:**
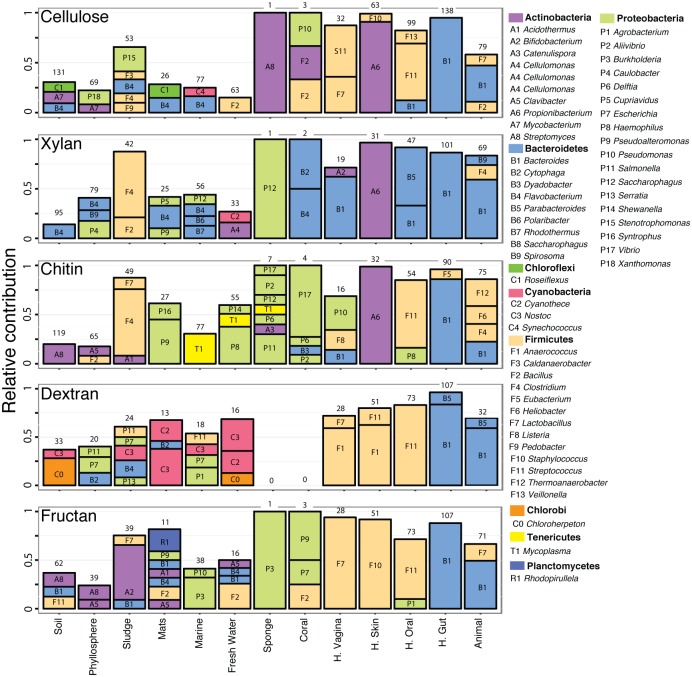
Relative contribution (%) of major potential degrader genera (i.e., >8% identified GH) to the pool of sequence targeting specific substrate, in each environment. Numbers represent the total number of identified bacterial genera endowed with potential to target the substrate.

Next, we defined microbial communities of degraders as the collection of identified bacterial genera associated with the potential to target cellulose, xylan, fructan, dextran, chitin, OAP, OPP, or Mixed substrate. In order to identify the degrader communities, we used the taxonomic annotation of the detected GH sequences. As expected [2], GH sequences for starch and oligosaccharides processing were associated with many genera. Traits for cellulose, xylan, and chitin were associated with tens to hundreds of genera. Finally the diversity of genera with the potential for metabolizing dextran and fructan was further reduced (Fig 2). The degrader community in human and animal metagenomes was strongly skewed toward few taxa from the Bacteroidetes, Actinobacterium, and Proteobacteria phyla. In both human gut and in animal samples, the pool of sequences for GH was dominated by sequence associated with *Bacteroides* whereas *Streptococcus* dominated in the human mouth, *Propionibacterium* in human skin, and *Lactobacillus* in human vagina. In corals and sponges, the few identified GH sequences were also derived from a reduced number of bacterial genera. In metagenomes from sludges, the community of degraders was moderately skewed toward few genera depending of the considered substrate (e.g., *Clostridium* for chitin and xylan). In the other environments the contribution of identified degraders to the pool of GH was more evenly distributed. Some of the identified degrader genera were detected in most ecosystem-types (e.g., *Bacteroidetes*, *Bacillus*) whereas some were restricted to specific environments (e.g., *Xylella*).

Across samples, sequences for the degrader community accounted for ~2 to ~82% (median value) of taxonomically identified sequences, in coral and vagina samples, respectively (S1 Fig). In addition, variation in the composition of the degrader community correlated with the composition of the non-degrader community (r_Spearman_ = 0.69, p = 0.001, S2 Fig). This suggested that the environmental parameters are affecting both the degraders and the non-degraders. However, the carbohydrate supply, being a major factor affecting microbial community composition in terrestrial ecosystems [11], is likely to act directly on the degrader community and indirectly on the non degraders through intergeneric association and competition [42].

Although similar numbers of degrader lineages are found across ecosystems, except in coral and sponge samples, host associated metagenomes displayed strong bias toward reduced number of degrader genera. These ecosystem-types constitute stable environments with constant supply of nutrient and little spatio-temporal variation. These stable and nutrient rich ecosystems promote the selection of specific lineages whereas “open” ecosystem-types, experiencing spatial and temporal variation of the nutrient supply harbor more diverse communities of degrader lineages [38]. This increased diversity likely results from spatial and temporal heterogeneity of open-environments and is likely to buffer the impact of fluctuating microbial community [43–45]. In contrast, in human and animal associated metagenomes, microbial communities are skewed towards few genera with increased GH-content and reduced genome size [46,47], thus increasing the overall frequency of GH sequences. In these communities, carbohydrate processing, and thus the entire environment functioning, is more vulnerable to perturbation affecting degraders [29,48,49].

Interestingly, in environments where the GH distribution and the assumed carbohydrate supply do not match, identifying the degrader lineages highlighted two trends. First, in the human vagina, the high frequency of GH32 and 68, targeting fructan, is associated with abundant *Lactobacillus* (phylum Firmicutes). These enzymes are potentially involved in the biosynthesis and metabolism of fructose-derived exopolysaccharides and biofilms [2,39,50]. Next, in human skin, the high frequency of cellulases matched with abundant GH5 found systematically in *Propionibacterium* (phylum Actinobacteria)[2,9]. Although secreted by *P*. *acnes* isolates [51], the exact function of these potential cellulases remains to be elucidated as the skin is not expected to contain large amount of cellulose. Thus, the prevalence of GH in a specific environment reflects the adaptation to nutrient supply, the requirement of GH for biosynthetic pathways (e.g., biofilms), and the phylogenetic conservatism of functional traits.

### Conservatism of GH across environments

Next, we essayed the conservatism of GH sequences in environmental potential degraders in order to test if the observed variation of the GH content across ecosystems mirrored the phylogeny or the environment. In total 493 identified bacterial genera with GH genes were identified. Most had the potential to degrade starch and oligosaccharides and just 77 major potential carbohydrate degraders were associated with GH for cellulose, xylan, fructan, dextran, chitin, OPP, OAP, and mixed substrates (when excluding rare genera, i.e. <0.2 SGE/metagenome) (S3 Fig). Most of these genera contained known degraders (e.g., *Clostridium*, *Xanthomonas*) [2,3,9]. In addition, several poorly-characterized genera were also identified (e.g., *Basfia*, *Novosphingobium*, *Leeuwenhoekiella*). Some degraders were cosmopolites (i.e., detected in most ecosystems, e.g., *Bacillus*, *Bacteroides*), some were intermediate cosmopolites, identified in few environments (e.g., *Caulobacter*), and few were restricted to specific environments (e.g., *Basfia*). Next, among the identified lineages, some were specialists with GH for a reduced number of carbohydrates (e.g., *Atopobium*, a vaginal commensal, and *Exiguobacterium*, an environmental cosmopolite) whereas some were generalists with the potential to target many substrates (e.g., *Bacteroides*, *Bacillus*, and *Streptomyces*)(S3 Fig).

**Fig 3:**
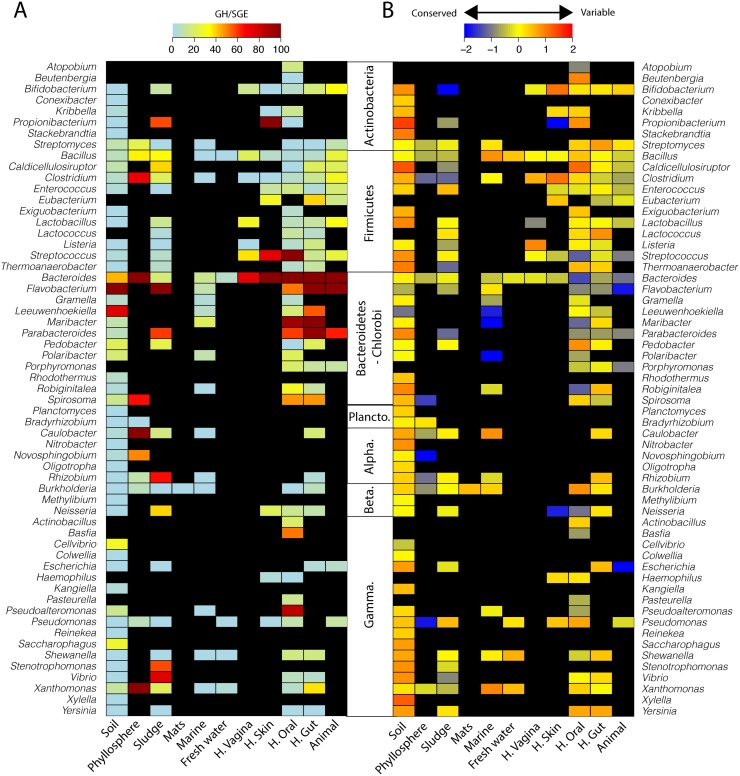
A, genus-specific frequency (per SGE) of sequences for GH targeting all carbohydrates but starch and oligosaccharides (median value) across environments. B, coefficient of variation of the genus-specific frequency of sequences for GH targeting all carbohydrates but starch and oligosaccharides. “Conserved” mirrors constant GH/SGE within ecosystem whereas “Variable” reflects variation of GH/SEG within ecosystem for each individual genus.

Among the major potential degraders, most cosmopolites and intermediate cosmopolites, except some Bacteroidetes, displayed conserved GH/SGE across environments (Fig 3A). This suggested that, in most genera, the phylogeny strongly affects the GH content and this supported the phylogenetic conservatisms of GH at the genus level in sequenced bacterial genomes [2,9]. Conversely, in variable *Bacteroides*, *Parabacteroides*, and *Flavobacterium*, the environment is likely strongly affecting the GH content. This suggested that, depending on the phylum, both the phylogeny and the environment could explain the lineage-specific GH content.

Thus, we next investigated the relative contribution of ecosystem and taxonomy on the genus specific GH content, across bacterial phyla (S4 Fig). In most phyla, the taxonomic origin, not the ecosystem, was a major source of variation of the potential for carbohydrate degradation (e.g., >40% of the observed variation in Fusobacteria and Planctomycetes). However in some phyla (e.g., Thermotogae and Tenericutes) the taxonomic affiliation accounted for <5% of observed GH/SGE variation. The environment-type and interactive effect between environment and taxonomy, also significantly affected the distribution of the GH in bacterial genera, accounting respectively for 1.5–17% and 0.7–13% of the observed variation (S4 Fig). Thus, overall, our data suggested that first the taxonomy, and the associate phylogeny, and next the environment affected the genus-specific GH content. This was further confirmed by the significant correlation between overall community composition and the variation in functional potential for carbohydrate processing across environments (n = 13 environment types, *r*_*mantel*_ = 0.42, p = 0.001) (Fig 1C, S5 Fig) and across samples (n = 1,934 metagenomes, *r*_*mantel*_ = 0.55, *p* = 0.001). Thus, despite variation across environments, the genus specific GH content is best described by the taxonomic affiliation of the considered lineages, at the genus level. Functional traits for carbohydrate processing are not randomly distributed among environmental bacterial genera.

### Connecting community structure and potential for carbohydrate deconstruction

**Fig 4:**
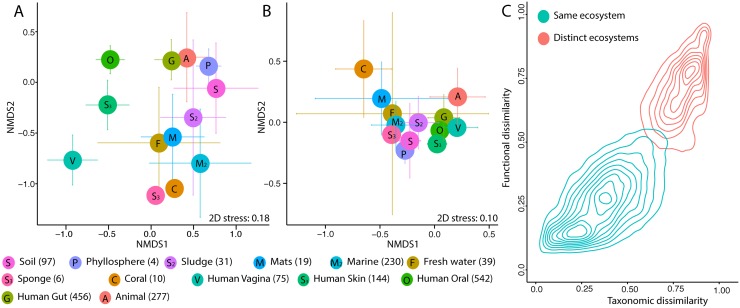
Non-metric multidimensional scaling ordination based on Bray-Curtis dissimilarities depicting the variation in frequency of sequences for GH targeting all carbohydrates except oligosaccharides and starch identified in microbial communities (A) and overall microbial communities composition (B), and color coded by environments (average/environment and SD, the number of datasets is in parentheses). C, Kernel density-plot for the relation between taxonomic and functional (based on identified GH sequences for all carbohydrate except oligosaccharides and starch) dissimilarities in pairs of communities.

Next, we investigated the connection between the overall microbial community composition and the potential for carbohydrate processing, across metagenomes (Fig 4). This analysis highlighted the taxonomic and functional proximity of microbiomes within most environments (Fig 4A and 4B). In addition, microbial communities from distinct environments but exposed to supposedly similar carbohydrates (e.g., animal vs. human gut), also overlapped structurally and functionally. This suggested that the overall microbial community composition and the potential for carbohydrate processing were linked. In order to test this connection, we assayed the dissimilarities in the potential for carbohydrate processing (*F*_*BC*_) and the overall taxonomic composition (*C*_*BC*_) across pairs of metagenomes (Fig 4C). First, even some completely different communities (i.e., *C*_*BC*_~1) shared potential for carbohydrate processing (i.e., *F*_*BC*_<1). This highlighted the central function of GH enzymes, their broad distribution across bacteria and environments [2,7,9] and converging functions in environmental communities regarding carbohydrate processing [52,53]. On the contrary, even taxonomically identical communities (i.e., *C*_*BC*_~0) displayed variation in their GH content (i.e., *F*_*BC*_>0). This suggested that, although conserved in most bacterial genera, closely related lineages (e.g., species) could possibly display variation of their potential for carbohydrate utilization [13]. Next, communities were more similar, compositionally and functionally, within the same environment than across environments. This supports ecosystem-specific GH composition (Figs 1 and 4A) and suggests that microbial community composition is a major factor affecting the overall potential for carbohydrate processing. Finally, within environments, compositional and functional dissimilarity correlated, the higher *F*_*BC*_ being associated with higher *C*_*BC*_.

### Prospectus

As described here, shotgun metagenomics provided a path to depict the taxonomy and functional potential for carbohydrate processing of complex environmental microbial communities. Nevertheless, many limitations have been associated with this technique [7,13]. Specifically, we recognize that, fungi and other microeukaryotes, although important members of microbial communities, were not included in this study. Second, accurate annotation of individual sequences in databases depends on the availability of biochemically-characterized homologs. GH are among the most characterized enzymes and their predicted substrate specificity was derived from biochemically characterized bacterial homologs [2,5]. However GH sometime display broader substrate specificity than described here and although GH are essential for carbohydrate processing, many other enzymes are involved in this process. Third, DNA extraction and sequencing procedures are known to affect the distribution of identified sequences. However, these bias were shown to have limited impact on discrimination of microbial communities from distinct environments [54]. These issues are invariably associated with metagenomics and can affect our conclusions in unknown direction. We also recognize that GH, although central for the processing of carbohydrates, are not the only CAZymes involves in this process. Indeed GH are known to act synergistically with other CAZymes (e.g., LPMO) and accessory domains (e.g., CBM) in order to fully deconstruct complex substrate (e.g., plant cell wall)[4,6].

Nevertheless, quantifying the distribution, the substrate specificity, and the taxonomic origin of sequences for glycoside hydrolases across 1,934 metagenomes provides an unprecedented opportunity for understanding organizing principles of the connection between community composition and the potential for carbohydrate processing, a key reaction in many environments [11]. First, a limited number of bacterial genera contribute to the pool of GH in the environment and their distribution produces ecosystem-specific potential for carbohydrate utilization. This reflects the limited distribution of genes for breaking down carbohydrate in bacterial lineages [2,9]. Across microbiomes, fluctuation in the community of the major degraders correlates with the non-degrader community thus confirming how important the carbohydrate supply is on the community of degraders [11].

As depicted here, the environment selects for both specific GH and specific lineages. In consequence, the assembly of microbial communities mirrors both deterministic and stochastic processes [55]. Indeed, in most ecosystems several ecologically similar, yet not identical, potential carbohydrate degraders can coexist and compete. This functional redundancy among degraders produces functionally similar but structurally distinct communities. Next, as suggested by Ferrenberg et al., stable microbial communities are more influenced by stochastic processes [55]. Finally, although conserved in most bacterial genera, some lineages may display variation of the GH content within genus [56]. Interspecific variation within these genera may result in variable overall functional potential with little variation in the community structure, when characterized at the genus level [57]. Together, these variations can influence the relation between potential for carbohydrate deconstruction and the overall microbial community. In consequence, the microbial community structure cannot be inferred from the identified potential for carbohydrate utilization. However, within ecosystems, the potential for carbohydrate utilization is highly conserved, relative to the overall microbial community structure. This suggests that environmental parameters, including carbohydrate supply [11], filter microbial lineages based on their potential for carbohydrate utilization. However the potential for carbohydrate utilization is constrained to specific lineages, at the genus level. In consequence, microbial community structure and function correlate and thus, knowing the microbial community composition (at the genus level), one could potentially infer the distribution of traits for carbohydrate utilization.

The phylogenetically conserved potential for polysaccharide utilization in bacterial genera detected in metagenomes in this study, and in sequenced bacterial genera [2,9] suggests that identifying the composition is essential to understand, and potentially predict, the distribution of genes involved in polysaccharide utilization in environmental microbial communities.

In the future, increasing the diversity of reference genomes will provide a better understanding of the phylogenetic distribution of genes for carbohydrate utilization, especially in poorly-characterized lineages (e.g., *Curtobacterium*, *Actinospica*)[6]. These lineages, even if poorly abundant, can contribute to the pool of GH [7], and thus might potentially affect the processing of carbohydrate, an essential reaction in many environments.

## Materials and Methods

### Metagenomic datasets

Publically accessible SEED-annotated metagenomic datasets (n = 1,934) were downloaded from the MG-RAST server, using the MG-RAST API (S1 Table) [35,58,59], and datasets were grouped by features and biomes according to the bioportal ontology (http://bioportal.bioontology.org/ontologies/). In order to identify all the sequences associated with GH in the samples, sequences for each GH/CBM family, as defined in the CAZy database [5], were extracted from the Pfam server and mapped against all sequenced genomes using SEED annotations [9,60]. SEED functional annotation of these traits was then used as a reference to investigate the SEED-annotated sequences provided by MG-RAST output files (i.e., XXX_650.Superblat.expand.protein) for functional annotations. The resulting hits and their corresponding sequences were then subjected to a Pfam_scan (analysis (PfamA 27.0 db, e-value<1×10^−5^) [61] to confirm functional annotations (S4 Table). This approach allowed us to identify short sequences from metagenomes matching GH from sequenced bacterial genomes. The taxonomy of the identified GH, and the overall community composition (at the genus level) for each dataset, was retrieved using taxonomic annotation of the corresponding sequences using M5nr database [59,62]

### GH substrate specificity

Glycoside hydrolases are among the most characterized enzymes. Many families have specific structure/function and display narrowed substrate specificity. GH families were assigned to substrate target categories according to the substrate specificities of characterized enzymes from bacteria, as stated in the CAZy database. GH families targeting cellulose, xylan, chitin, starch (and glycogen), fructan, dextran, and oligosaccharides were identified [2,5,8,9]. Some GH families were identified as targeting Other Plant Polysaccharides (i.e., polysaccharides other than cellulose, xylan, starch, fructan), Other Animal Polysaccharides (i.e., polysaccharides other than starch-glycogen, chitin), and Mixed when targeting several substrates (S4 Table).

### Statistics

Statistical analyses were performed using ‘Stat’ (v3.3.0) and ‘Vegan’ (v2.4–1) packages in the R software environment (v3.3.0) [63,64]. For clustering of environments, we summarized the data (i.e., we computed the median frequency of GH sequences per sequenced genome equivalent (SGE), the GH composition, and to community composition) by environment type. Then Bray-Curtis dissimilarities between pairs of environments were computed and the clustering was achieved by hierarchical clustering (S6 Fig). For clustering based on the GH composition, we first selected metagenomic datasets containing at least 500 identified GH sequences, then the GH distribution was rarefied and dissimilarity was computed using Bray-Curtis index. Noteworthy, none of the datasets from Sponge or Coral was included in the analysis. Finally, for the clustering according to the community composition, datasets with more than 10,000 taxonomically identified hits were considered (no dataset from Coral could be included in this test). Correlation between environment comparisons was achieved by running Mantel correlation test (999 permutations) [63] on the corresponding distance matrices.

The contribution of genera to the pool of GH sequences was achieved by analyzing the taxonomic origin (at the genus level) of identified GH sequences [2]. Then sequences for enzymes targeting specific substrate (S2 Table) were tallied by environment and by genus. Then, the total number of bacterial genera endowed with the potential to target the substrate was obtained. Major degrader genera were arbitrarily determined, for clarity of purpose, as bacterial genera contributing at least 8% of the identified GH for a considered substrate, in at least one specific environment.

The impact of environment and taxonomy, and the associated phylogeny, on genus specific GH content was identified in bacterial genera in datasets with at least one genus-specific sequenced genome equivalent (i.e., 3Mbp). Next, we computed the median value for each GH family, in each genus, in each environment, per sequenced genome equivalent. Finally, we ran a PERMANOVA (GH~Environment*Genus, with 500 permutations)[63], for each phylum. The results are expressed as percent estimated variance explained by genus, environment, and the interaction of genus by environment (S5 Fig).

## Supporting Information

S1 Fig Relative contribution of all sequences from potential carbohydrate degraders to the entire pool of sequences across ecosystems. (PDF)

S2 Fig Bray-Curtis dissimilarity in communities of potential degraders and non-degraders, among pairs of metagenomes. (PDF)

S3 Fig A, genus-specific frequency (per SGE) of sequences for GH in potential degraders (average value) across datasets. B, coefficient of variation of the genu- 515 specific frequency of sequences for GH.
(PDF)

S4 Fig Relative contribution of environment and taxonomy on the variation of potential for carbohydrate utilization in all identified bacterial genera, per phylum. In parentheses are number of identified genera and the number environments where these genera were detected, respectively. Plotted values are proportional to the estimates of the variance components, all p<0.05. (*Phyla for which the number of identified genera and/or environments was too small to evaluate the combined effect of environment by genus).
(PDF)

S5 Fig Environments clustering based on GH frequency (GH/SGE), overall community composition (identified at the genus level), and GH distribution. Correlation between clustering investigated using Mantel-test (n_permutations_ = 999).
(PDF)

S1 Table Metagenomic datasets, from MG-RAST, included in this study. (DOCX)

S2 Table Tukey Post-hoc test (substrate across ecosystems, *P*>0.05). (DOCX)

S3 Table Tukey Post-hoc test (substrate by ecosystem, *P*>0.05). (DOCX)

S4 Table Glycoside Hydrolases (GHs), with identified PFam id, and the corresponding targeted substrate. “Other Plant Polysaccharides” and “Other Animal Polysaccharides” are used for GH family targeting substrates not previously identified, and derived for plant or animal. GHs with mixed substrates are enzymes associated with multiple substrates.
(DOCX)
