# Supplementary material for: Glycoside Hydrolases across Environmental Microbial Communities
Source: PLoS Comput Biol. 2016 Dec 19;12(12):e1005300. doi: 10.1371/journal.pcbi.1005300 (PMC5218504; doi:10.1371/journal.pcbi.1005300)

S1 Figure. Relative contribution of all sequences from potential carbohydrate degraders to the entire pool of sequences across ecosystems.

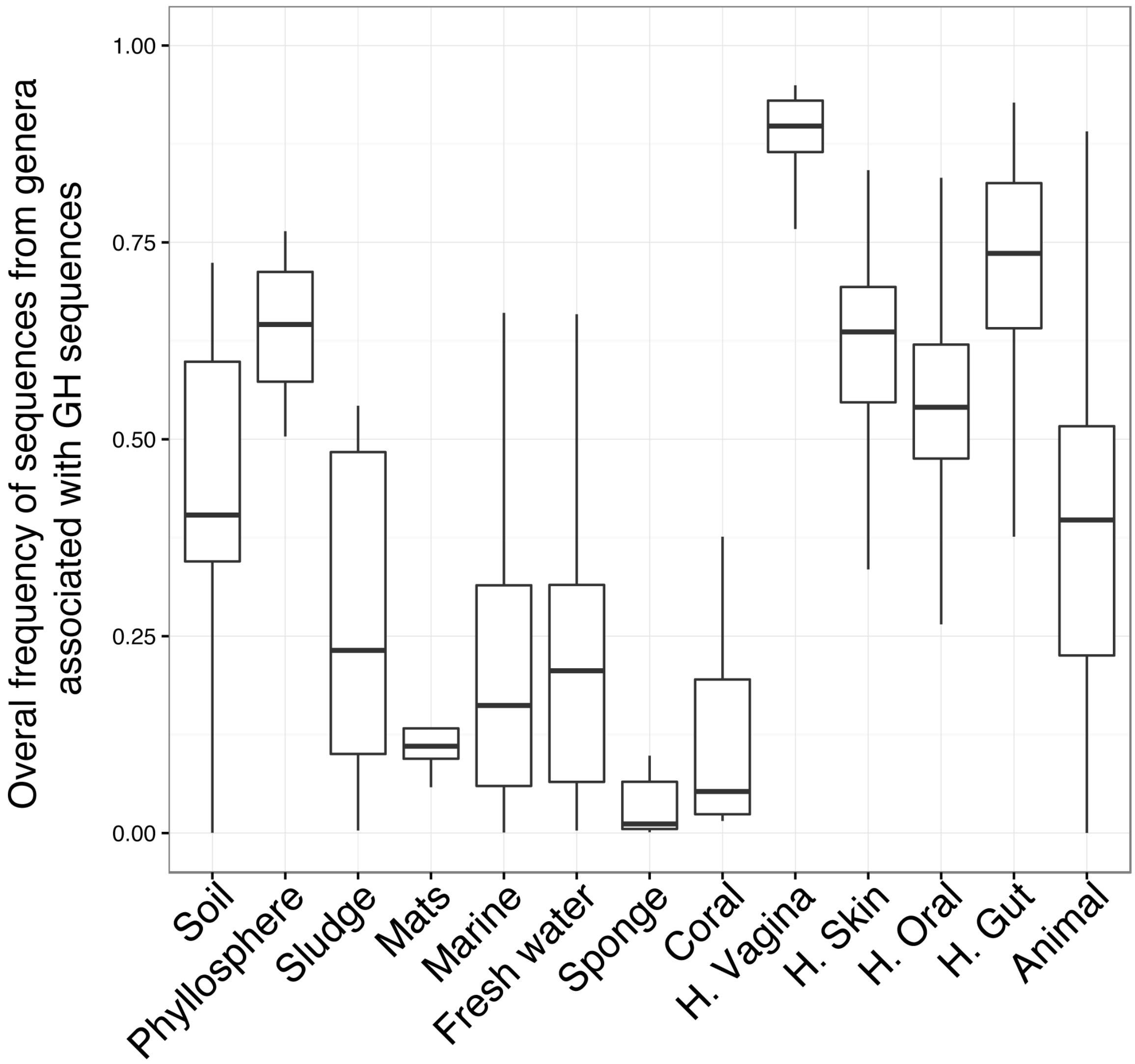

Supplement: S1 Fig — (PDF) [file pcbi.1005300.s001.pdf]
