# Supplementary material for: Glycoside Hydrolases across Environmental Microbial Communities
Source: PLoS Comput Biol. 2016 Dec 19;12(12):e1005300. doi: 10.1371/journal.pcbi.1005300 (PMC5218504; doi:10.1371/journal.pcbi.1005300)

S2 Figure. Bray-Curtis dissimilarity in communities of potential degraders and non-degraders, among pairs of metagenomes

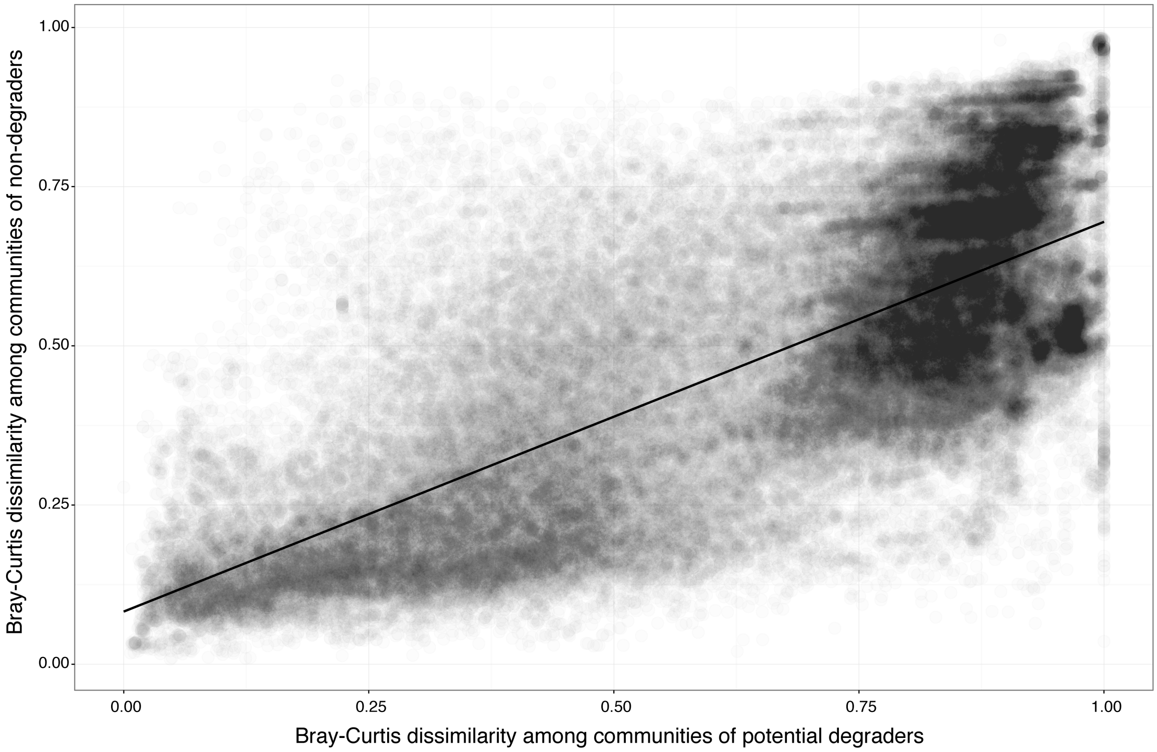

Supplement: S2 Fig — (PDF) [file pcbi.1005300.s002.pdf]
