# Supplementary material for: Glycoside Hydrolases across Environmental Microbial Communities
Source: PLoS Comput Biol. 2016 Dec 19;12(12):e1005300. doi: 10.1371/journal.pcbi.1005300 (PMC5218504; doi:10.1371/journal.pcbi.1005300)

S3 Figure. A, genus-specific frequency (per SGE) of sequences for GH in potential degraders (average value) across datasets. B, coefficient of variation of the genu- 515 specific frequency of sequences for GH.

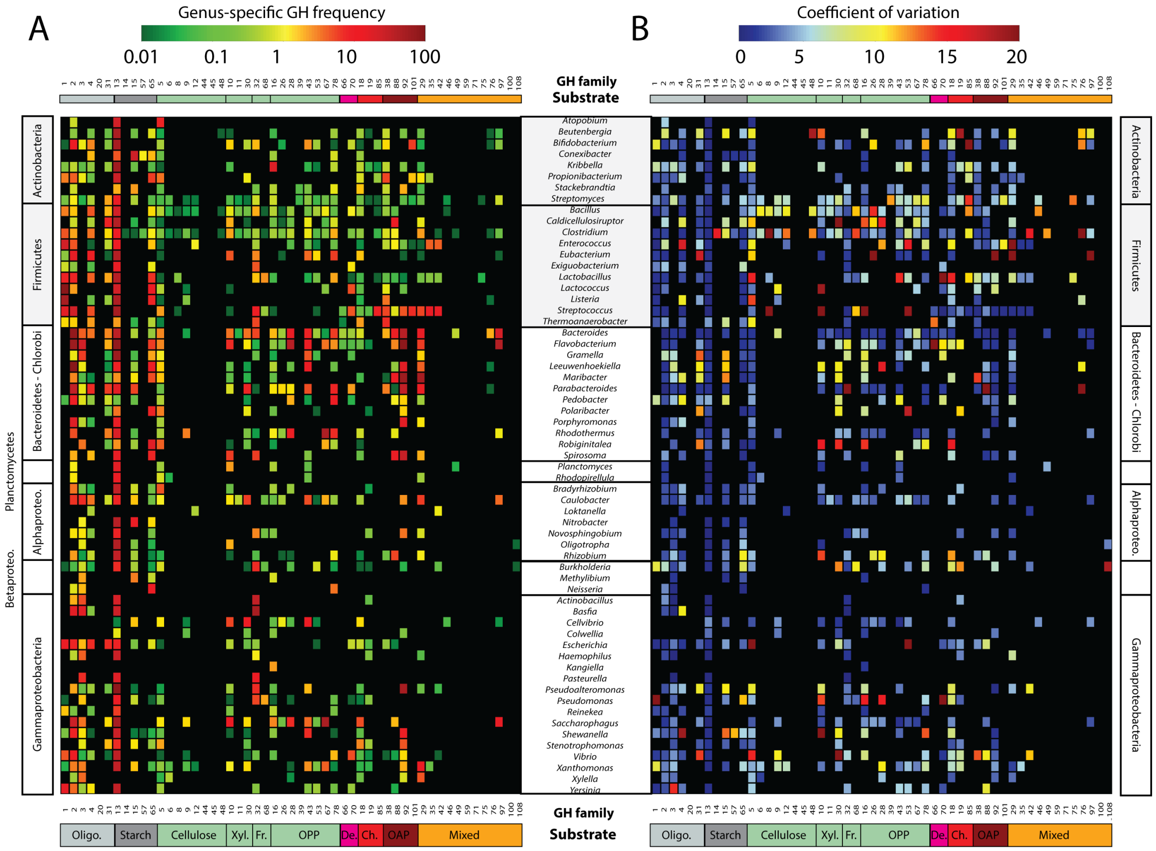

Supplement: S3 Fig — A, genus-specific frequency (per SGE) of sequences for GH in potential degraders (average value) across datasets. B, coefficient of variation of the genu- 515 specific frequency of sequences for GH. (PDF) [file pcbi.1005300.s003.pdf]
