# Supplementary material for: Glycoside Hydrolases across Environmental Microbial Communities
Source: PLoS Comput Biol. 2016 Dec 19;12(12):e1005300. doi: 10.1371/journal.pcbi.1005300 (PMC5218504; doi:10.1371/journal.pcbi.1005300)

S4 Figure. Relative contribution of environment and taxonomy on the variation of potential for carbohydrate utilization in all identified bacterial genera, per phylum. In parentheses are number of identified genera and the number environments where these genera were detected, respectively. Plotted values are proportional to the estimates of the variance components, all $p<0.05$. (*Phyla for which the number of identified genera and/or environments was too small to evaluate the combined effect of environment by genus).

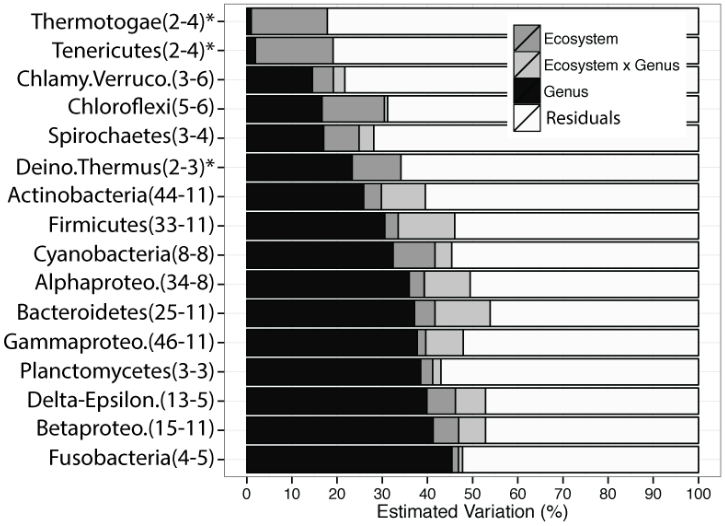

Supplement: S4 Fig — In parentheses are number of identified genera and the number environments where these genera were detected, respectively. Plotted values are proportional to the estimates of the variance components, all p<0.05. (*Phyla for which the number of identified genera and/or environments was too small to evaluate the combined effect of environment by genus). (PDF) [file pcbi.1005300.s004.pdf]
