# Supplementary material for: Glycoside Hydrolases across Environmental Microbial Communities
Source: PLoS Comput Biol. 2016 Dec 19;12(12):e1005300. doi: 10.1371/journal.pcbi.1005300 (PMC5218504; doi:10.1371/journal.pcbi.1005300)

S5 Figure. Environments clustering based on GH frequency (GH/SGE), overall community composition (identified at the genus level), and GH distribution. Correlation between clustering investigated using Mantel-test ($n_{permutations}$=999).

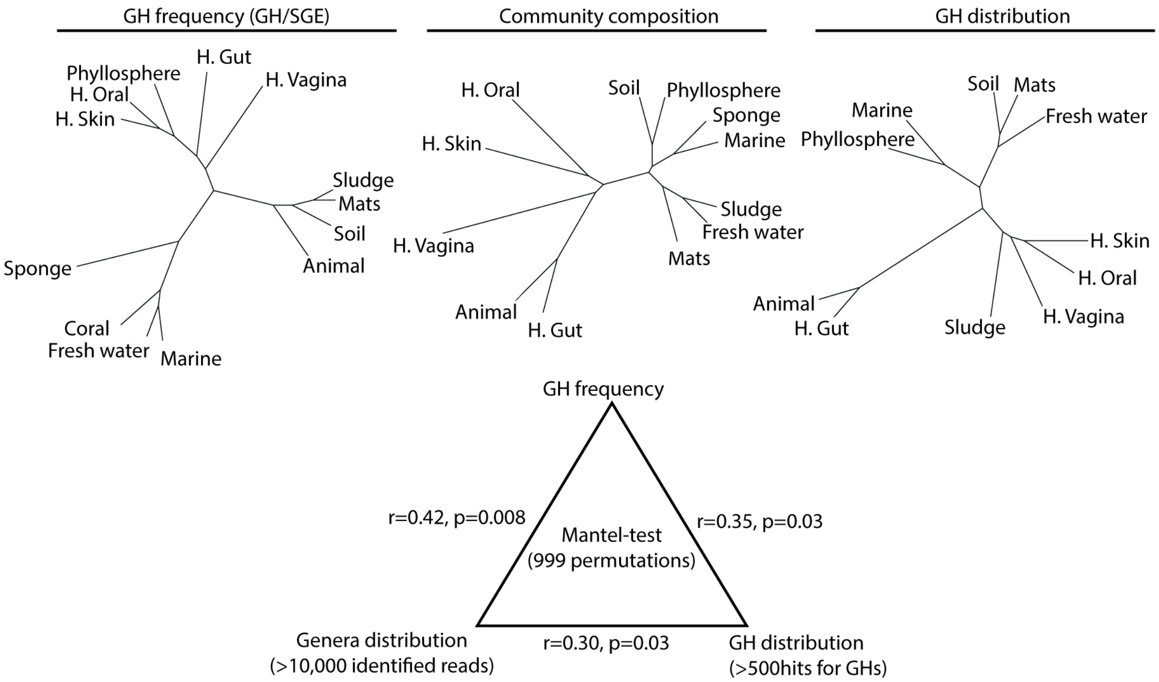

Supplement: S5 Fig — Correlation between clustering investigated using Mantel-test (npermutations = 999). (PDF) [file pcbi.1005300.s005.pdf]
