# Supplementary material for: Glycoside Hydrolases across Environmental Microbial Communities
Source: PLoS Comput Biol. 2016 Dec 19;12(12):e1005300. doi: 10.1371/journal.pcbi.1005300 (PMC5218504; doi:10.1371/journal.pcbi.1005300)
Supplement: S1 Table — (DOCX) [file pcbi.1005300.s006.docx]

S1 Table. Metagenomic datasets, from MG-RAST, included in this study.

| Ecosystem | Metagenome ID (MG-RAST) |
| --- | --- |
| Coral | mgm4440370, mgm4440371, mgm4440372, mgm4440373, mgm4440374, mgm4440375, mgm4440376, mgm4440377, mgm4440379, mgm4440381 |
| Sponge | mgm4461451, mgm4461452, mgm4461453, mgm4461454, mgm4461455, mgm4461456 |
| Phyllosphere | mgm4447810, mgm4447811, mgm4449956, mgm4450328 |
| Mat | mgm4440963, mgm4440964, mgm4440965, mgm4440966, mgm4440967, mgm4440968, mgm4440969, mgm4440970, mgm4440971, mgm4440972, mgm4443746, mgm4443747, mgm4443749, mgm4443750, mgm4443762, mgm4445126, mgm4445129, mgm4447951, mgm4448052 |
| Soil | mgm4443231, mgm4443232, mgm4445803, mgm4445806, mgm4445990, mgm4445993, mgm4445994, mgm4446153, mgm4449126, mgm4449252, mgm4449255, mgm4449256, mgm4449284, mgm4449356, mgm4449357, mgm4449358, mgm4449359, mgm4449360, mgm4449362, mgm4449363, mgm4449364, mgm4449365, mgm4449877, mgm4450123, mgm4450125, mgm4450126, mgm4450127, mgm4450729, mgm4450731, mgm4450750, mgm4450752, mgm4451103, mgm4451104, mgm4451105, mgm4451106, mgm4453246, mgm4453247, mgm4453254, mgm4453256, mgm4453257, mgm4453261, mgm4453274, mgm4453406, mgm4453407, mgm4453433, mgm4453434, mgm4453435, mgm4453436, mgm4461011, mgm4461013, mgm4461840, mgm4465556, mgm4465558, mgm4477803, mgm4477804, mgm4477805, mgm4477872, mgm4477874, mgm4477875, mgm4477876, mgm4477877, mgm4477899, mgm4477901, mgm4477903, mgm4489643, mgm4489645, mgm4489648, mgm4489649, mgm4489654, mgm4489655, mgm4489656, mgm4502539, mgm4502540, mgm4502541, mgm4502542, mgm4502543, mgm4502923, mgm4502924, mgm4502925, mgm4502926, mgm4502927, mgm4502928, mgm4502929, mgm4502930, mgm4502931, mgm4502932, mgm4502933, mgm4502934, mgm4502935, mgm4504797, mgm4504798, mgm4508937, mgm4508938, mgm4508939, mgm4508940, mgm4508941, mgm4508942 |
| Fresh-water | mgm4440055, mgm4440056, mgm4440059, mgm4440063, mgm4440064, mgm4440065, mgm4440066, mgm4440281, mgm4440282, mgm4441102, mgm4441137, mgm4441138, mgm4441590, mgm4441663, mgm4441695, mgm4441696, mgm4443679, mgm4443680, mgm4443681, mgm4443682, mgm4443683, mgm4443684, mgm4449206, mgm4453064, mgm4453083, mgm4460441, mgm4460448, mgm4460449, mgm4461585, mgm4461675, mgm4467029, mgm4467058, mgm4467059, mgm4470602, mgm4478241, mgm4478242, mgm4516288, mgm4516289, mgm4516290 |
| Sludge/Waste | mgm4441092, mgm4441093, mgm4443753, mgm4443754, mgm4443755, mgm4443756, mgm4480719, mgm4480764, mgm4480776, mgm4480815, mgm4480859, mgm4480861, mgm4480863, mgm4480864, mgm4480865, mgm4480867, mgm4493725, mgm4493726, mgm4493727, mgm4494621, mgm4494623, mgm4501098, mgm4501099, mgm4508401, mgm4508402, mgm4508403, mgm4508404, mgm4508805, mgm4508831, mgm4521514, mgm4521534 |
| Marine | mgm4440036, mgm4440037, mgm4440038, mgm4440039, mgm4440040, mgm4440041, mgm4440061, mgm4440067, mgm4440212, mgm4440213, mgm4440275, mgm4440276, mgm4440279, mgm4440280, mgm4440320, mgm4440321, mgm4440329, mgm4440330, mgm4440358, mgm4440359, mgm4440360, mgm4440361, mgm4440362, mgm4440363, mgm4440364, mgm4440365, mgm4440960, mgm4440961, mgm4440973, mgm4440984, mgm4441020, mgm4441021, mgm4441022, mgm4441025, mgm4441041, mgm4441050, mgm4441051, mgm4441055, mgm4441056, mgm4441057, mgm4441062, mgm4441121, mgm4441125, mgm4441126, mgm4441127, mgm4441129, mgm4441132, mgm4441133, mgm4441134, mgm4441135, mgm4441136, mgm4441139, mgm4441143, mgm4441144, mgm4441145, mgm4441146, mgm4441147, mgm4441148, mgm4441149, mgm4441150, mgm4441151, mgm4441152, mgm4441153, mgm4441155, mgm4441156, mgm4441167, mgm4441214, mgm4441215, mgm4441347, mgm4441363, mgm4441568, mgm4441570, mgm4441573, mgm4441574, mgm4441582, mgm4441585, mgm4441589, mgm4441591, mgm4441593, mgm4441594, mgm4441595, mgm4441596, mgm4441597, mgm4441598, mgm4441599, mgm4441600, mgm4441601, mgm4441602, mgm4441603, mgm4441604, mgm4441605, mgm4441606, mgm4441607, mgm4441608, mgm4441609, mgm4441610, mgm4441611, mgm4441613, mgm4441614, mgm4441615, mgm4441616, mgm4441617, mgm4441618, mgm4441621, mgm4441622, mgm4441623, mgm4441624, mgm4441625, mgm4441626, mgm4441627, mgm4441628, mgm4441629, mgm4441661, mgm4441662, mgm4442451, mgm4442464, mgm4442466, mgm4442467, mgm4442498, mgm4442499, mgm4442500, mgm4442503, mgm4442582, mgm4442626, mgm4442708, mgm4442709, mgm4443685, mgm4443686, mgm4443687, mgm4443688, mgm4443689, mgm4443691, mgm4443693, mgm4443695, mgm4443697, mgm4443698, mgm4443699, mgm4443700, mgm4443701, mgm4443702, mgm4443703, mgm4443704, mgm4443705, mgm4443706, mgm4443707, mgm4443708, mgm4443709, mgm4443711, mgm4443712, mgm4443713, mgm4443714, mgm4443715, mgm4443716, mgm4443717, mgm4443718, mgm4443719, mgm4443720, mgm4443721, mgm4443722, mgm4443723, mgm4443724, mgm4443725, mgm4443726, mgm4443729, mgm4443731, mgm4443732, mgm4443733, mgm4443734, mgm4443765, mgm4443766, mgm4444863, mgm4446341, mgm4446342, mgm4446411, mgm4448226, mgm4449104, mgm4453374, mgm4453375, mgm4453379, mgm4453380, mgm4453381, mgm4453382, mgm4454501, mgm4454502, mgm4454503, mgm4454505, mgm4454506, mgm4454686, mgm4454687, mgm4454688, mgm4454689, mgm4454691, mgm4454693, mgm4454694, mgm4454695, mgm4454696, mgm4454698, mgm4454699, mgm4454701, mgm4454702, mgm4454703, mgm4455295, mgm4459940, mgm4459941, mgm4461444, mgm4463936, mgm4487624, mgm4487625, mgm4491344, mgm4491346, mgm4491347, mgm4491348, mgm4492532, mgm4494598, mgm4494599, mgm4494600, mgm4494601, mgm4494602, mgm4494603, mgm4494604, mgm4494605, mgm4494606, mgm4494607, mgm4494608, mgm4494609, mgm4503511, mgm4503529, mgm4516291, mgm4516292, mgm4516293, mgm4524607 |
| Animal | mgm4440052, mgm4440053, mgm4440054, mgm4440283, mgm4440284, mgm4440285, mgm4440286, mgm4440319, mgm4440463, mgm4440464, mgm4441679, mgm4441680, mgm4441681, mgm4441682, mgm4442701, mgm4444164, mgm4444165, mgm4444702, mgm4444703, mgm4445755, mgm4448367, mgm4450678, mgm4450679, mgm4450680, mgm4453653, mgm4461341, mgm4461342, mgm4461343, mgm4461344, mgm4461345, mgm4461346, mgm4461347, mgm4461348, mgm4461349, mgm4461350, mgm4461351, mgm4461352, mgm4461353, mgm4461354, mgm4461355, mgm4461356, mgm4461357, mgm4461358, mgm4461360, mgm4461361, mgm4461362, mgm4461363, mgm4461364, mgm4461365, mgm4461366, mgm4461367, mgm4461368, mgm4461369, mgm4461370, mgm4461371, mgm4461372, mgm4461374, mgm4461375, mgm4461376, mgm4461377, mgm4461378, mgm4461379, mgm4461380, mgm4461383, mgm4491686, mgm4492788, mgm4492790, mgm4492791, mgm4492794, mgm4492795, mgm4492797, mgm4492799, mgm4492800, mgm4492801, mgm4492802, mgm4492803, mgm4492805, mgm4492808, mgm4492809, mgm4492810, mgm4492811, mgm4492812, mgm4492815, mgm4492816, mgm4492817, mgm4492819, mgm4492820, mgm4492821, mgm4492824, mgm4492825, mgm4492826, mgm4492828, mgm4492830, mgm4492831, mgm4492833, mgm4492834, mgm4492835, mgm4492836, mgm4492837, mgm4492838, mgm4492839, mgm4492840, mgm4492841, mgm4492845, mgm4492847, mgm4492848, mgm4492849, mgm4492850, mgm4492851, mgm4492852, mgm4492853, mgm4492854, mgm4492855, mgm4492856, mgm4492858, mgm4492859, mgm4492860, mgm4492863, mgm4492864, mgm4492865, mgm4492871, mgm4492872, mgm4492873, mgm4492874, mgm4492876, mgm4492878, mgm4492879, mgm4492880, mgm4492881, mgm4492882, mgm4492884, mgm4492885, mgm4492886, mgm4492887, mgm4492888, mgm4492890, mgm4492891, mgm4492892, mgm4492893, mgm4492894, mgm4492895, mgm4492896, mgm4492898, mgm4492899, mgm4492900, mgm4492902, mgm4492904, mgm4492905, mgm4492908, mgm4492909, mgm4492910, mgm4492911, mgm4492912, mgm4492964, mgm4492966, mgm4492967, mgm4492970, mgm4492972, mgm4492973, mgm4492974, mgm4492975, mgm4492976, mgm4517704, mgm4517705, mgm4517706, mgm4517707, mgm4517708, mgm4517709, mgm4517710, mgm4517711, mgm4517712, mgm4517713, mgm4517714, mgm4517715, mgm4517716, mgm4517717, mgm4517718, mgm4517719, mgm4517720, mgm4517721, mgm4517722, mgm4517723, mgm4517724, mgm4517725, mgm4517726, mgm4517727, mgm4517728, mgm4517729, mgm4517730, mgm4517731, mgm4517732, mgm4517733, mgm4517734, mgm4517735, mgm4517736, mgm4517737, mgm4517738, mgm4517739, mgm4517740, mgm4517741, mgm4517742, mgm4517743, mgm4517744, mgm4517745, mgm4519873, mgm4519874, mgm4519885, mgm4519886, mgm4519887, mgm4519888, mgm4519889, mgm4519890, mgm4519891, mgm4519892, mgm4519893, mgm4519894, mgm4519895, mgm4519896, mgm4519897, mgm4519898, mgm4520058, mgm4520059, mgm4520060, mgm4520061, mgm4520062, mgm4520063, mgm4520064, mgm4520065, mgm4520066, mgm4520067, mgm4520068, mgm4520069, mgm4520070, mgm4520071, mgm4520072, mgm4520073, mgm4520074, mgm4520075, mgm4520076, mgm4520077, mgm4520078, mgm4520079, mgm4520080, mgm4520081, mgm4520082, mgm4520083, mgm4520084, mgm4520085, mgm4520086, mgm4520087, mgm4520088, mgm4520091, mgm4520100, mgm4520103, mgm4520104, mgm4520105, mgm4520106, mgm4520135, mgm4520137, mgm4525800, mgm4525802, mgm4525818, mgm4525822, mgm4525824, mgm4525828, mgm4525831, mgm4525832, mgm4525833, mgm4525834, mgm4525835, mgm4525836, mgm4525838, mgm4525839, mgm4525840, mgm4525842, mgm4525844, mgm4525845, mgm4525846, mgm4525848, mgm4525849 |
| Human gut | mgm4440610, mgm4440611, mgm4440613, mgm4440614, mgm4440615, mgm4440616, mgm4440639, mgm4440640, mgm4440823, mgm4440824, mgm4440825, mgm4440826, mgm4440939, mgm4440941, mgm4440942, mgm4440943, mgm4440944, mgm4440945, mgm4440946, mgm4440947, mgm4440948, mgm4440949, mgm4440950, mgm4440951, mgm4461119, mgm4461121, mgm4461122, mgm4461123, mgm4461124, mgm4461125, mgm4461126, mgm4461127, mgm4461128, mgm4461129, mgm4461130, mgm4461131, mgm4461132, mgm4461133, mgm4461134, mgm4461135, mgm4461136, mgm4461137, mgm4461138, mgm4461139, mgm4461140, mgm4461141, mgm4461142, mgm4461143, mgm4461144, mgm4461145, mgm4461146, mgm4461147, mgm4461148, mgm4461149, mgm4461150, mgm4461151, mgm4461152, mgm4461153, mgm4461154, mgm4461155, mgm4461156, mgm4461157, mgm4461158, mgm4461159, mgm4461160, mgm4461161, mgm4461162, mgm4461163, mgm4461164, mgm4461165, mgm4461166, mgm4461167, mgm4461168, mgm4461169, mgm4461170, mgm4461171, mgm4461172, mgm4461173, mgm4461174, mgm4461175, mgm4461176, mgm4461177, mgm4461178, mgm4461179, mgm4461180, mgm4461181, mgm4461182, mgm4461183, mgm4461184, mgm4461185, mgm4461186, mgm4461187, mgm4461188, mgm4461189, mgm4461190, mgm4461191, mgm4461192, mgm4461193, mgm4461194, mgm4461195, mgm4461196, mgm4461197, mgm4461198, mgm4461199, mgm4461200, mgm4461201, mgm4461202, mgm4461203, mgm4461204, mgm4461205, mgm4461206, mgm4461207, mgm4461208, mgm4461209, mgm4461210, mgm4461211, mgm4461212, mgm4461213, mgm4461214, mgm4461215, mgm4461216, mgm4461217, mgm4461218, mgm4461219, mgm4461220, mgm4461221, mgm4461222, mgm4461223, mgm4461224, mgm4461225, mgm4461226, mgm4461227, mgm4461228, mgm4461229, mgm4461284, mgm4461285, mgm4461286, mgm4461287, mgm4461288, mgm4461289, mgm4461290, mgm4461291, mgm4461292, mgm4461293, mgm4461294, mgm4461295, mgm4461296, mgm4461297, mgm4461298, mgm4461299, mgm4461300, mgm4461301, mgm4466296, mgm4472090, mgm4472091, mgm4472092, mgm4472093, mgm4472094, mgm4472095, mgm4472096, mgm4472097, mgm4472098, mgm4472099, mgm4472100, mgm4472101, mgm4472102, mgm4472103, mgm4472104, mgm4472105, mgm4472117, mgm4472124, mgm4472125, mgm4472128, mgm4472129, mgm4472130, mgm4472131, mgm4472132, mgm4472133, mgm4472136, mgm4472137, mgm4472138, mgm4472139, mgm4472140, mgm4472141, mgm4472142, mgm4472143, mgm4472152, mgm4472153, mgm4472158, mgm4472159, mgm4472164, mgm4472165, mgm4472176, mgm4472177, mgm4472184, mgm4472185, mgm4472191, mgm4472194, mgm4472195, mgm4472196, mgm4472197, mgm4472198, mgm4472199, mgm4472214, mgm4472215, mgm4472223, mgm4472248, mgm4472249, mgm4472250, mgm4472251, mgm4472264, mgm4472265, mgm4472270, mgm4472275, mgm4472276, mgm4472277, mgm4472278, mgm4472300, mgm4472301, mgm4472302, mgm4472303, mgm4472330, mgm4472331, mgm4472332, mgm4472333, mgm4472336, mgm4472337, mgm4472342, mgm4472343, mgm4472346, mgm4472347, mgm4472356, mgm4472357, mgm4472360, mgm4472361, mgm4472362, mgm4472363, mgm4472376, mgm4472377, mgm4472386, mgm4472387, mgm4472392, mgm4472393, mgm4472394, mgm4472395, mgm4472402, mgm4472403, mgm4472405, mgm4472408, mgm4472411, mgm4472412, mgm4472413, mgm4472421, mgm4472425, mgm4472430, mgm4472447, mgm4472448, mgm4472453, mgm4472460, mgm4472461, mgm4472464, mgm4472465, mgm4472466, mgm4472467, mgm4472470, mgm4472471, mgm4472472, mgm4472473, mgm4472476, mgm4472477, mgm4472478, mgm4472479, mgm4472482, mgm4472483, mgm4472518, mgm4472519, mgm4472550, mgm4472551, mgm4472559, mgm4472560, mgm4472561, mgm4472562, mgm4472563, mgm4472564, mgm4472565, mgm4472566, mgm4472567, mgm4472568, mgm4472569, mgm4472570, mgm4472571, mgm4472572, mgm4472573, mgm4472574, mgm4472575, mgm4472576, mgm4472577, mgm4472578, mgm4472579, mgm4472580, mgm4472581, mgm4472582, mgm4472583, mgm4472584, mgm4472585, mgm4472586, mgm4472589, mgm4472590, mgm4472591, mgm4472593, mgm4472595, mgm4472596, mgm4472597, mgm4472598, mgm4472599, mgm4472600, mgm4472602, mgm4472603, mgm4472604, mgm4472607, mgm4472610, mgm4472611, mgm4472612, mgm4472613, mgm4472614, mgm4472615, mgm4472616, mgm4472618, mgm4472619, mgm4472620, mgm4472627, mgm4472628, mgm4472629, mgm4472630, mgm4472631, mgm4472633, mgm4472634, mgm4472636, mgm4472637, mgm4472641, mgm4472643, mgm4472644, mgm4472645, mgm4472646, mgm4472650, mgm4472651, mgm4472654, mgm4472655, mgm4472658, mgm4472661, mgm4472664, mgm4472665, mgm4472666, mgm4472668, mgm4472672, mgm4472675, mgm4472676, mgm4472677, mgm4472680, mgm4472681, mgm4472682, mgm4472700, mgm4472701, mgm4472720, mgm4472724, mgm4472766, mgm4472770, mgm4472771, mgm4472774, mgm4472783, mgm4472808, mgm4472872, mgm4472878, mgm4472882, mgm4472883, mgm4472896, mgm4472899, mgm4472924, mgm4472926, mgm4472934, mgm4472935, mgm4472936, mgm4472937, mgm4472939, mgm4472945, mgm4472947, mgm4472948, mgm4472950, mgm4473008, mgm4473009, mgm4473012, mgm4473014, mgm4473021, mgm4473023, mgm4473026, mgm4473041, mgm4473042, mgm4473048, mgm4473059, mgm4473074, mgm4473075, mgm4473084, mgm4473086, mgm4473087, mgm4473089, mgm4473129, mgm4473156, mgm4473157, mgm4473248, mgm4473299, mgm4473302, mgm4473317, mgm4473324, mgm4473351, mgm4473362, mgm4473366, mgm4473380, mgm4473383, mgm4473393, mgm4473399, mgm4473402, mgm4473403, mgm4489630, mgm4489632, mgm4489633, mgm4489635, mgm4489636, mgm4489637, mgm4489661, mgm4489663, mgm4489665, mgm4489667, mgm4491401, mgm4491403, mgm4491404, mgm4491406, mgm4491407, mgm4491410, mgm4491412, mgm4491414, mgm4491417, mgm4491418, mgm4491419, mgm4491421, mgm4491423, mgm4491477, mgm4491479, mgm4491482, mgm4491487, mgm4491488, mgm4491562, mgm4508944, mgm4508945, mgm4508946, mgm4508947, mgm4508948 |
| Human Vagina | mgm4472424, mgm4472435, mgm4472436, mgm4472441, mgm4472449, mgm4472450, mgm4472454, mgm4472500, mgm4472501, mgm4472502, mgm4472503, mgm4472520, mgm4472521, mgm4472534, mgm4472535, mgm4472552, mgm4472553, mgm4472601, mgm4472621, mgm4472625, mgm4472626, mgm4472632, mgm4472635, mgm4472642, mgm4472647, mgm4472652, mgm4472653, mgm4472692, mgm4472693, mgm4472694, mgm4472706, mgm4472709, mgm4472710, mgm4472712, mgm4472713, mgm4472714, mgm4472715, mgm4472725, mgm4472730, mgm4472736, mgm4472737, mgm4472739, mgm4472744, mgm4472745, mgm4472748, mgm4472785, mgm4472796, mgm4472866, mgm4472867, mgm4472929, mgm4472931, mgm4472992, mgm4473001, mgm4473018, mgm4473033, mgm4473035, mgm4473036, mgm4473050, mgm4473051, mgm4473054, mgm4473072, mgm4473190, mgm4473202, mgm4473217, mgm4473218, mgm4473220, mgm4473241, mgm4473242, mgm4473245, mgm4473254, mgm4473265, mgm4473266, mgm4473333, mgm4473341, mgm4473345 |
| Human Skin | mgm4472358, mgm4472370, mgm4472399, mgm4472474, mgm4472475, mgm4472490, mgm4472491, mgm4472504, mgm4472505, mgm4472506, mgm4472507, mgm4472510, mgm4472511, mgm4472522, mgm4472523, mgm4472536, mgm4472537, mgm4472540, mgm4472541, mgm4472544, mgm4472545, mgm4472546, mgm4472547, mgm4472649, mgm4472707, mgm4472711, mgm4472717, mgm4472718, mgm4472723, mgm4472728, mgm4472731, mgm4472733, mgm4472741, mgm4472743, mgm4472746, mgm4472747, mgm4472749, mgm4472750, mgm4472757, mgm4472761, mgm4472763, mgm4472768, mgm4472769, mgm4472772, mgm4472776, mgm4472780, mgm4472787, mgm4472788, mgm4472790, mgm4472791, mgm4472800, mgm4472801, mgm4472809, mgm4472810, mgm4472813, mgm4472815, mgm4472816, mgm4472818, mgm4472823, mgm4472824, mgm4472826, mgm4472827, mgm4472831, mgm4472832, mgm4472833, mgm4472834, mgm4472838, mgm4472841, mgm4472844, mgm4472845, mgm4472846, mgm4472847, mgm4472849, mgm4472850, mgm4472855, mgm4472858, mgm4472860, mgm4472861, mgm4472863, mgm4472870, mgm4472901, mgm4472902, mgm4472905, mgm4472906, mgm4472909, mgm4472910, mgm4472911, mgm4472914, mgm4472918, mgm4472920, mgm4472946, mgm4472952, mgm4472953, mgm4472965, mgm4472967, mgm4472970, mgm4472973, mgm4472975, mgm4473032, mgm4473038, mgm4473056, mgm4473057, mgm4473062, mgm4473063, mgm4473068, mgm4473069, mgm4473077, mgm4473078, mgm4473080, mgm4473081, mgm4473083, mgm4473096, mgm4473098, mgm4473099, mgm4473102, mgm4473103, mgm4473118, mgm4473138, mgm4473139, mgm4473141, mgm4473150, mgm4473151, mgm4473153, mgm4473160, mgm4473162, mgm4473163, mgm4473165, mgm4473166, mgm4473171, mgm4473183, mgm4473205, mgm4473206, mgm4473221, mgm4473232, mgm4473236, mgm4473239, mgm4473250, mgm4473262, mgm4473269, mgm4473290, mgm4473296, mgm4473321, mgm4473335, mgm4473384 |
| Human Oral | mgm4444195, mgm4444196, mgm4472108, mgm4472109, mgm4472112, mgm4472113, mgm4472114, mgm4472115, mgm4472120, mgm4472121, mgm4472122, mgm4472123, mgm4472126, mgm4472127, mgm4472146, mgm4472147, mgm4472148, mgm4472149, mgm4472156, mgm4472157, mgm4472160, mgm4472166, mgm4472167, mgm4472173, mgm4472178, mgm4472179, mgm4472188, mgm4472189, mgm4472200, mgm4472201, mgm4472202, mgm4472203, mgm4472206, mgm4472207, mgm4472210, mgm4472211, mgm4472216, mgm4472217, mgm4472220, mgm4472221, mgm4472224, mgm4472225, mgm4472228, mgm4472229, mgm4472234, mgm4472235, mgm4472236, mgm4472237, mgm4472244, mgm4472245, mgm4472252, mgm4472253, mgm4472254, mgm4472255, mgm4472258, mgm4472259, mgm4472266, mgm4472267, mgm4472281, mgm4472282, mgm4472285, mgm4472286, mgm4472287, mgm4472288, mgm4472289, mgm4472290, mgm4472291, mgm4472293, mgm4472295, mgm4472296, mgm4472297, mgm4472298, mgm4472299, mgm4472305, mgm4472307, mgm4472308, mgm4472311, mgm4472312, mgm4472313, mgm4472314, mgm4472315, mgm4472316, mgm4472326, mgm4472327, mgm4472328, mgm4472329, mgm4472334, mgm4472335, mgm4472338, mgm4472339, mgm4472340, mgm4472344, mgm4472345, mgm4472348, mgm4472349, mgm4472352, mgm4472353, mgm4472354, mgm4472355, mgm4472364, mgm4472365, mgm4472367, mgm4472368, mgm4472369, mgm4472372, mgm4472373, mgm4472374, mgm4472375, mgm4472378, mgm4472379, mgm4472380, mgm4472381, mgm4472384, mgm4472385, mgm4472390, mgm4472391, mgm4472396, mgm4472397, mgm4472400, mgm4472401, mgm4472406, mgm4472407, mgm4472409, mgm4472410, mgm4472419, mgm4472422, mgm4472426, mgm4472428, mgm4472431, mgm4472432, mgm4472433, mgm4472434, mgm4472437, mgm4472438, mgm4472439, mgm4472440, mgm4472442, mgm4472443, mgm4472444, mgm4472445, mgm4472446, mgm4472451, mgm4472452, mgm4472455, mgm4472456, mgm4472457, mgm4472458, mgm4472459, mgm4472462, mgm4472463, mgm4472468, mgm4472469, mgm4472480, mgm4472481, mgm4472484, mgm4472485, mgm4472486, mgm4472487, mgm4472488, mgm4472489, mgm4472492, mgm4472493, mgm4472494, mgm4472495, mgm4472497, mgm4472498, mgm4472499, mgm4472508, mgm4472509, mgm4472512, mgm4472513, mgm4472514, mgm4472515, mgm4472516, mgm4472517, mgm4472524, mgm4472525, mgm4472528, mgm4472529, mgm4472530, mgm4472531, mgm4472532, mgm4472533, mgm4472538, mgm4472539, mgm4472542, mgm4472543, mgm4472548, mgm4472549, mgm4472554, mgm4472555, mgm4472556, mgm4472557, mgm4472558, mgm4472587, mgm4472588, mgm4472592, mgm4472594, mgm4472605, mgm4472606, mgm4472608, mgm4472609, mgm4472617, mgm4472622, mgm4472623, mgm4472624, mgm4472638, mgm4472639, mgm4472640, mgm4472648, mgm4472656, mgm4472657, mgm4472659, mgm4472660, mgm4472662, mgm4472663, mgm4472667, mgm4472669, mgm4472670, mgm4472671, mgm4472673, mgm4472674, mgm4472678, mgm4472679, mgm4472683, mgm4472684, mgm4472685, mgm4472686, mgm4472687, mgm4472688, mgm4472689, mgm4472690, mgm4472691, mgm4472695, mgm4472696, mgm4472697, mgm4472698, mgm4472699, mgm4472703, mgm4472704, mgm4472705, mgm4472708, mgm4472716, mgm4472719, mgm4472721, mgm4472722, mgm4472726, mgm4472727, mgm4472729, mgm4472732, mgm4472734, mgm4472735, mgm4472738, mgm4472740, mgm4472742, mgm4472751, mgm4472752, mgm4472753, mgm4472754, mgm4472755, mgm4472756, mgm4472758, mgm4472759, mgm4472760, mgm4472762, mgm4472764, mgm4472767, mgm4472773, mgm4472775, mgm4472777, mgm4472778, mgm4472779, mgm4472781, mgm4472782, mgm4472784, mgm4472786, mgm4472789, mgm4472792, mgm4472793, mgm4472794, mgm4472795, mgm4472797, mgm4472798, mgm4472799, mgm4472802, mgm4472803, mgm4472804, mgm4472805, mgm4472806, mgm4472807, mgm4472811, mgm4472812, mgm4472814, mgm4472817, mgm4472819, mgm4472820, mgm4472821, mgm4472822, mgm4472825, mgm4472828, mgm4472829, mgm4472830, mgm4472835, mgm4472836, mgm4472837, mgm4472839, mgm4472840, mgm4472842, mgm4472843, mgm4472848, mgm4472851, mgm4472852, mgm4472853, mgm4472854, mgm4472856, mgm4472859, mgm4472862, mgm4472864, mgm4472865, mgm4472868, mgm4472869, mgm4472871, mgm4472873, mgm4472875, mgm4472876, mgm4472877, mgm4472879, mgm4472880, mgm4472881, mgm4472884, mgm4472885, mgm4472886, mgm4472887, mgm4472888, mgm4472890, mgm4472891, mgm4472892, mgm4472893, mgm4472894, mgm4472895, mgm4472897, mgm4472898, mgm4472900, mgm4472903, mgm4472904, mgm4472907, mgm4472908, mgm4472912, mgm4472913, mgm4472915, mgm4472916, mgm4472917, mgm4472919, mgm4472921, mgm4472922, mgm4472923, mgm4472925, mgm4472927, mgm4472928, mgm4472930, mgm4472932, mgm4472933, mgm4472940, mgm4472941, mgm4472942, mgm4472944, mgm4472949, mgm4472951, mgm4472954, mgm4472955, mgm4472956, mgm4472957, mgm4472958, mgm4472959, mgm4472960, mgm4472961, mgm4472962, mgm4472963, mgm4472966, mgm4472968, mgm4472969, mgm4472971, mgm4472972, mgm4472974, mgm4472976, mgm4472977, mgm4472978, mgm4472980, mgm4472981, mgm4472983, mgm4472984, mgm4472986, mgm4472987, mgm4472989, mgm4472990, mgm4472991, mgm4472993, mgm4473002, mgm4473003, mgm4473005, mgm4473006, mgm4473011, mgm4473015, mgm4473017, mgm4473020, mgm4473024, mgm4473027, mgm4473029, mgm4473030, mgm4473039, mgm4473044, mgm4473045, mgm4473047, mgm4473053, mgm4473060, mgm4473065, mgm4473066, mgm4473071, mgm4473090, mgm4473092, mgm4473093, mgm4473095, mgm4473101, mgm4473105, mgm4473106, mgm4473108, mgm4473109, mgm4473111, mgm4473112, mgm4473114, mgm4473115, mgm4473117, mgm4473120, mgm4473121, mgm4473123, mgm4473124, mgm4473126, mgm4473127, mgm4473130, mgm4473132, mgm4473133, mgm4473135, mgm4473136, mgm4473142, mgm4473144, mgm4473145, mgm4473147, mgm4473148, mgm4473154, mgm4473159, mgm4473168, mgm4473169, mgm4473172, mgm4473174, mgm4473175, mgm4473177, mgm4473178, mgm4473180, mgm4473181, mgm4473184, mgm4473186, mgm4473187, mgm4473189, mgm4473192, mgm4473193, mgm4473195, mgm4473196, mgm4473198, mgm4473199, mgm4473201, mgm4473203, mgm4473208, mgm4473209, mgm4473211, mgm4473212, mgm4473214, mgm4473215, mgm4473223, mgm4473224, mgm4473226, mgm4473227, mgm4473229, mgm4473230, mgm4473233, mgm4473251, mgm4473257, mgm4473259, mgm4473260, mgm4473263, mgm4473271, mgm4473272, mgm4473275, mgm4473277, mgm4473278, mgm4473281, mgm4473284, mgm4473287, mgm4473289, mgm4473292, mgm4473293, mgm4473303, mgm4473306, mgm4473309, mgm4473311, mgm4473312, mgm4473315, mgm4473318, mgm4473327, mgm4473330, mgm4473336, mgm4473338, mgm4473339, mgm4473342, mgm4473348, mgm4473350, mgm4473354, mgm4473357, mgm4473360, mgm4473363, mgm4473369, mgm4473372, mgm4473375, mgm4473377, mgm4473378, mgm4473381, mgm4473387, mgm4473390, mgm4473392, mgm4473395, mgm4473396, mgm4473406, mgm4501142, mgm4501143, mgm4501145, mgm4501146, mgm4501147, mgm4501148, mgm4501149, mgm4501150, mgm4501151, mgm4501159, mgm4501161 |
