# Supplementary material for: Glycoside Hydrolases across Environmental Microbial Communities
Source: PLoS Comput Biol. 2016 Dec 19;12(12):e1005300. doi: 10.1371/journal.pcbi.1005300 (PMC5218504; doi:10.1371/journal.pcbi.1005300)
Supplement: S2 Table — (DOCX) [file pcbi.1005300.s007.docx]

S2 Table. Tukey Post-hoc test (substrate across ecosystems, *P*>0.05)

|  | All | Oligo | Starch | Poly | Cel | Xyl | Fru | OPP | Dex | Chi | OAP | M |
| --- | --- | --- | --- | --- | --- | --- | --- | --- | --- | --- | --- | --- |
| Soil | C | D | D | C | B | B | D | B | DE | B | C | C |
| Phyllosphere | BC | BCD | CD | BC | AB | AB | D | AB | CDE | AB | BC | BC |
| Sludge | C | D | D | C | B | B | D | B | DE | B | C | C |
| Mats | C | D | D | C | B | BC | D | B | E | B | C | C |
| Marine | C | D | D | C | B | C | D | B | E | B | C | C |
| Fresh water | C | D | D | C | B | C | D | B | DE | B | C | C |
| Sponge | C | D | D | C | B | C | D | B | E | B | C | C |
| Coral | C | D | D | C | B | C | D | B | E | B | C | C |
| H. Vagina | A | A | A | B | B | C | A | B | E | B | C | A |
| H. Skin | B | BC | C | B | A | B | C | B | C | A | B | B |
| H. Oral | B | B | B | B | B | C | B | B | A | B | B | A |
| H. Gut | A | A | C | A | B | A | C | A | B | A | A | AB |
| Animal | C | CD | D | C | B | B | D | B | CD | B | C | C |
