# Supplementary material for: Glycoside Hydrolases across Environmental Microbial Communities
Source: PLoS Comput Biol. 2016 Dec 19;12(12):e1005300. doi: 10.1371/journal.pcbi.1005300 (PMC5218504; doi:10.1371/journal.pcbi.1005300)
Supplement: S3 Table — (DOCX) [file pcbi.1005300.s008.docx]

S3 Table. Tukey Post-hoc test (substrate by ecosystem, *P*>0.05).

|  | Soil | Phyllosphere | Sludge | Mats | Marine | Fresh water | Sponge | Coral | H. Vagina | H. Skin | H. Oral | H. Gut | Animal |
| --- | --- | --- | --- | --- | --- | --- | --- | --- | --- | --- | --- | --- | --- |
| Cellulose | C | BC | BC |  | BC | AB |  |  | B | B | DE | D | C |
| Xylan | AB | ABC | AB |  | BCD | AB |  |  | B | C | EF | C | BC |
| Fructan | C | C | BC |  | CD | B |  |  | A | C | C | D | C |
| OPP | A | A | A |  | B | AB |  |  | B | C | EF | A | A |
| Dextran | C | C | C |  | D | B |  |  | B | C | D | D | C |
| Chitin | C | C | BC |  | AB | A |  |  | B | C | F | D | C |
| OAP | BC | AB | BC |  | B | AB |  |  | B | A | B | A | A |
| Mixed | AB | ABC | AB |  | A | A |  |  | A | AB | A | B | B |
