# Supplementary material for: Glycoside Hydrolases across Environmental Microbial Communities
Source: PLoS Comput Biol. 2016 Dec 19;12(12):e1005300. doi: 10.1371/journal.pcbi.1005300 (PMC5218504; doi:10.1371/journal.pcbi.1005300)
Supplement: S4 Table — “Other Plant Polysaccharides” and “Other Animal Polysaccharides” are used for GH family targeting substrates not previously identified, and derived for plant or animal. GHs with mixed substrates are enzymes associated with multiple substrates. (DOCX) [file pcbi.1005300.s009.docx]

S4 Table. Glycoside Hydrolases (GHs), with identified PFam id, and the corresponding targeted substrate. “Other Plant Polysaccharides” and “Other Animal Polysaccharides” are used for GH family targeting substrates not previously identified, and derived for plant or animal. GHs with mixed substrates are enzymes associated with multiple substrates.

| GH family | Pfam | Substrate |
| --- | --- | --- |
| GH1 | PF00232 | Oligosaccharides |
| GH2 | PF00703  PF02836  PF02837 | Oligosaccharides |
| GH3 | PF00933  PF01915 | Oligosaccharides |
| GH4 | PF02056  PF11975 | Oligosaccharides |
| GH5 | PF00150 | Cellulose |
| GH6 | PF01341 | Cellulose |
| GH8 | PF01270 | Cellulose |
| GH9 | PF00759 | Cellulose |
| GH10 | PF00331 | Xylan |
| GH11 | PF00457 | Xylan |
| GH12 | PF01670 | Cellulose |
| GH13 | PF00128 | Starch/Glycogen |
| GH14 | PF01373 | Starch/Glycogen |
| GH15 | PF00723 | Starch/Glycogen |
| GH16 | PF00722 | Other Plant Polysaccharides |
| GH17 | PF00332 | Other Plant Polysaccharides |
| GH18 | PF00704 | Chitin |
| GH19 | PF00182 | Chitin |
| GH20 | PF00728 | Oligosaccharides |
| GH25 | PF01183 | Lysozyme |
| GH26 | PF02156 | Other Plant Polysaccharides |
| GH28 | PF00295 | Other Plant Polysaccharides |
| GH29 | PF01120 | Mixed Polysaccharides |
| GH30 | PF02055 | Xylan |
| GH31 | PF01055 | Oligosaccharides |
| GH32 | PF08244  PF00251 | Fructan |
| GH35 | PF01301 | Mixed Polysaccharides |
| GH38 | PF01074  PF07748 | Other Animal Polysaccharides |
| GH39 | PF01229 | Other Plant Polysaccharides |
| GH42 | PF02449  PF08533  PF08532 | Mixed Polysaccharides |
| GH43 | PF04616 | Other Plant Polysaccharides |
| GH44 | PF12891 | Cellulose |
| GH45 | PF02015 | Cellulose |
| GH46 | PF01374 | Mixed Polysaccharides |
| GH48 | PF02011 | Cellulose |
| GH49 | PF03718 | Mixed Polysaccharides |
| GH53 | PF07745 | Other Plant Polysaccharides |
| GH57 | PF03065 | Starch/Glycogen |
| GH59 | PF02057 | Mixed Polysaccharides |
| GH65 | PF03633  PF03632  PF03636 | Starch/Glycogen |
| GH66 | PF13199 | Dextran |
| GH67 | PF07477  PF07488  PF03648 | Other Plant Polysaccharides |
| GH68 | PF02435 | Fructan |
| GH70 | PF02324 | Dextran |
| GH71 | PF03659 | Mixed Polysaccharides |
| GH75 | PF07335 | Mixed Polysaccharides |
| GH76 | PF03663 | Mixed Polysaccharides |
| GH78 | PF05592 | Other Plant Polysaccharides |
| GH85 | PF03644 | Chitin |
| GH88 | PF07470 | Other Animal Polysaccharides |
| GH92 | PF07971 | Other Animal Polysaccharides |
| GH97 | PF10566 | Mixed Polysaccharides |
| GH100 | PF12899 | Mixed Polysaccharides |
| GH101 | PF12905 | Other Animal Polysaccharides |
| GH108 | PF05838 | Mixed Polysaccharides |
